# Thermo-fluid characteristics and exergy analysis of a twisted tube helical coil

**DOI:** 10.1038/s41598-024-78164-1

**Published:** 2024-11-13

**Authors:** Mahmoud Abdelmagied

**Affiliations:** Department of Refrigeration and Air Conditioning Technology, Faculty of Technology and Education, Helwan University, 11282 Cairo, Egypt

**Keywords:** Twisted flow, Passive technique, Heat transfer enhancement, Energy science and technology, Engineering

## Abstract

In the present investigation, the exergy of an innovative technique involving the integration of curved helical tubes with twisted passages was experimentally presented. This technique aims to improve the thermofluid characteristics by involving the swirl intensity of fluid flow in a twisted tube helical coil (*TTHC*). Six identical geometries with different pitch ratios $\Upsilon$ of 36 mm, 54 mm, and ∞ (smooth/no twisted) were experimentally explored at two different inner tube profiles of triangular and square cross-sections in counter flow arrangements. The experimental runs were carried out at 10,300 ≤ *Re*_*i*_ ≤ 37,800 and 2400 ≤ *Re*_*o*_ ≤17,600 for both the inner and annulus fluids, respectively. The results showed that the Nusselt number, *Nu*, increased by 39.6% and 41.5% for triangular and square inner twisted cross-section profiles, respectively, at a $\it \Upsilon$ of 36 mm at the expense of increasing *f* by 37.6% and 60.7%, respectively. The results also showed that the thermal performance factor reached 1.3 and 1.25 for a $\it \Upsilon$ of 36 mm for the triangular and square inner twisted tube profiles, respectively. A comprehensive study is performed to analyze the *TTHC* from thermal, frictional, and exergetic viewpoints. New correlations for expecting the annulus *Nu*_*o*_ and *f*_*o*_ are presented.

## Introduction

Compared to the flow in a straight tube, the fluid flow in a coiled tube is more complex. This can be attributed to the occurrence of secondary flow, which is generated by the centrifugal force. When the fluid is pumped into a curved tube, the fluid particle moves toward the outer surface of the tube, and secondary flow occurs. This secondary flow is expected to diminish the viscous and thermal boundary layer thicknesses and enhance the heat transfer rate.

A twisted tube helical coil (*TTHC*) can be considered a compact heat exchanger design. This process can be referred to as providing heat transfer surface area compared to a smooth surface area; moreover, tube corrugation increases fluid turbulence and decreases the viscous boundary layer. The new design is expected to provide high thermal stress flexibility and play a main role in various engineering applications, such as heat transfer, cold and freezing storage, air conditioning systems, power plants, chemical reactors, food processing, and saltwater desalination^1–3^. A *TTHC* provides a super heat transfer rate, flow resistance, and higher performance due to the secondary flow generated during motion in a curved tube beside the corrugated flow. Table 1 summarizes the reviews of utilizing twisted tubes in heat transfer and fluid flow applications. Zachár^4^ performed a numerical study to improve the heat transfer rate of coiled tubes with spiral corrugated walls. Zhang et al.^5^ experimentally achieved steam condensation in horizontally twisted elliptical tubes. Rainieri et al.^6^ investigated heat transfer in both straight and curved tubes experimentally at 5 < *Re* < 13 for glycerol and at 150 < *Re* < 1500 for ethylene glycol. This study verified the effectiveness of this approach when highly viscous fluids were treated. The heat transfer in a helical-ribbed tube with double twisted tape inserts and a smooth tube was described by Promvonge et al.^7^. Correlations between *Nu* and friction factor *f* were proposed. Farnam et al.^8^ showed intensified single-phase laminar flow forced convective heat transfer with a helical twisted tube in coil heat exchangers. The RSM was employed by Wang et al.^9^ to design a numerical model with different parameters for a novel outward helical corrugated tube. Omidi et al.^10^ investigated the laminar heat transfer and flow characteristics of helical coils with different lobed cross-sections. A correlation for predicting *Nu* is predicted. Kurnia and Sasmito^11^ numerically achieved heat transfer and entropy generation for different cross-sections of helical and straight tubes at constant wall heat flux. The laminar heat transfer and pressure drop flow characteristics of five different types of compound inserts in ducts were studied by Emani et al.^12^. New correlations for predicting *Nu* and *f* were developed. Dong et al.^13^ experimentally investigated the heat transfer and flow resistance characteristics of high-viscosity thermal oil and water in a spiral twisted tube.

**Table 1:** Review of the previous studies.

Authors, year	Flow regime, base fluid	Heat exchanger	Parameter	Results
Zachár^4^	Laminar and transitional flow regimes, numerical, water and ethylene glycol mixture	Helically tube	Different Inner diameter of the helical tubes, corrugation pitches, corrugation depths, and two working fluids.	The helical corrugated tube shows an increase of 80–100% in the heat transfer rate and an increase of 10–600% in pressure drop compared to the common helical design. New correlation was proposed to predict for the inner side heat transfer
Zhang et al.^5^	Turbulent flow, experimental, water	Straight tube	Different twisted pitches from 104 to 192 mm corresponding to ellipticity tube from0.71 to 0.86 as well as smooth tube	Average enhancement factors range from 0.87 to 1.34. The largest ellipticity of 0.86 present the highest condensation heat transfer coefficient by 34%. When the ellipticity exceeds 0.77, the twist pitch of 192 mm provides a better condensation performance than the smooth tube.
Rainieri et al.^6^	Laminar flow, experimental, Glycerol and Ethylene Glycol	Straight and coiled tubes	Straight and coiled tubes	The corrugation enhances heat transfer rate at higher *Re* range while the smooth helical coils enhance heat transfer rates at low *Re*.
Promvonge et al.^7^	Turbulent flow, experimental, water	Helical-ribbed tube with twisted tape inserts	Different twist ratio from 2.17 to 9.39	The co-swirling inserted tube performs much better than the ribbed/ smooth tube alone at a similar operating condition. The co-swirl tube at *Y* ≈ 8 yields the highest thermal performance at lower *Re*. Correlations of *Nu*, and *f* as functions of *Re*,* Pr* and *Y* are proposed.
Farnam et al.^8^	Laminar flow, experimental and numerical, water	Smooth and twisted helical tube	Different level of twisted helical design parameters and smooth helical tube.	The enhancement reached 14.2% and 7.7% for *Nu* and *f* for the medium level. The maximum performance index reaches 1.98 at the helical diameter of 50 mm corresponding to *Re* = 900.
Wang et al.^9^	Turbulent flow, numerical, water	Helically corrugated tubes	Different corrugation pitch ratio and corrugation height- ratio.	*Re* had the most effect on *Nu*, and its sensitivity coefficient is five times higher than that for the corrugation pitch ratio and corrugation height ratio.
Omidi et al.^10^	Laminar flow, numerical, water	Helical coil	Four lobed cross sections, different geometrical parameters (coil pitch, height, and diameter) and different fluids	A coil with *n* = 6 presents the highest *Nu* and the lowest friction *f*. The coil diameter has the greatest effect in comparison to the other geometrical parameters. The effect of adding Al_2_O_3_ nanoparticles to water increased *Nu* by 28% compared to water.
Kurnia and Sasmito^11^	Laminar flow, numerical, water	Helical coil and straight tubes	Various circular, ellipse, and square cross-sections	A helical tube provides higher heat transfer at the expense of higher pressure. The entropy generation in helical tubes is lower compared to straight tubes. The square cross-section obtained the highest heat transfer and pressure drop and entropy generation for both straight and helical tubes.
Emani et al.^12^	Laminar flow, experimental, water	Helical tube	Wire-coil diameter, wire-coil helix angle, tooth angle and tooth horizontal length of the screw tape and the twisted tape, corrugation height and pitch	Correlations for *Nu* and *f* were developed and a performance evaluation was done.
Dong et al.^13^	Turbulent flow, experimental, thermal oil and water.	Spiral twisted tube	Mass fluxes, the water and oil inlet temperature, and the heat transfer and flow resistance properties	The *h/Δp*,* h/(Δpq*_*v*_*)*, and *Nu/f* of the twisted tube were compared with that of two tube-shell heat exchangers. The fitting correlations of the *Nu*,* f* of the twisted tube were obtained. The twisted tube demonstrates an obvious heat transfer enhancement effect
Wang et al.^14^	Turbulent flow, experimental and numerical, water	Helically coiled-twisted trilobal tube	Different cross-section sizes and coil designs.	The HCTTT performs better in the hydrodynamic and thermal performance than that HCET, HCPT, and HCTT. Compared to the HCPT, the increment ratio on *Nu* of HCTTT is more than 19–31%, while the augmentation ratio of flow resistance is up to 24-38%.
Chang et al.^15^	Turbulent flow, numerical, water	Helical coils with square twisted section	Five twisted helical coils with different twist pitches, of 3, 4, 5, 6 and ∞.	The swirl enhancement dominates *Nu* and *f* augmentations for twisted helical coils. Relative to the untwisted coil, 19% of *Nu* with 69.8% *f* elevations are attainable by twisting channel. *Nu*, and *f* correlations of twisted helical coils are devised.
Yu et al.^16^	Turbulent flow, numerical, water	Twisted oval tube	Different geometric shape, direction of rotation and the orientations of wire coil’s cross section.	The wire coil with cross-section Et3 presents the highest thermo-hydraulic performance. The direction of the wire coil affects the thermo-hydraulic performance of the twisted tube significantly. The average increase ratio of *Nu* is 45.92% and the averaged *f* is 674.86%
Yang et al.^17^	Turbulent flow, numerical water	Smooth and spirally corrugated tubes	Different pitch ratio from 3.50:4.50, and corrugation depth ratio from 0.09:0.22 at *Re* from 10,000:35,000.	The heat transfer coefficient and turbulent kinetic energy confirm the superiority of the case with pitch ratio = 3.75, while the maximum augmentation of 258%. The pitch ratio of 4.5 generates less pressure loss, which is 1–8% larger than the smooth tube. The highest performance index at pitch ratio = 4.5 is reach 1.1 times than the lowest value. At corrugation depth ratio = 0.09, the hybrid smooth and spirally corrugated tube has better thermo-hydraulic performance.
Qian et al.^18^	Turbulent flow, numerical, water	Multi-start spirally corrugated tubes	Six different multi-start spirally corrugated tubes	The eight-start spirally corrugated tube is the highest heat transfer enhancement. The eight-spirally corrugated tube has a lower friction factor, which is 1.52–1.75 times the smooth tube. The performance index values of multi-start spirally corrugated tubes vary from 0.77 to 1.28 times compared to smooth tubes.
Aliabadi and Feizabadi^19^	Laminar flow, numerical, water	Twisted-tape and twisted tube	Different enhanced geometries and different levels of twist-pitches	The results showed that the performance improves as the twist-pitch is increased for all models and the maximum performance index reaches 3.21 at *Re* = 1800.
Reddy et al.^20^	Laminar flow, numerical, water	Helical tube-in-tube	Different configurations of the inner tube	*Nu* and *f* at different angular positions were evaluated. The hexagon geometry increases *Nu* by 17.05% and *f* by 15% compared with a circular tube at a Dean number of 400.
Yang et al.^21^	Turbulent flow, numerical, supercritical CO_2_	Helical tube with non-circular cross section	characteristic parameters pitch diameter and radius	With increasing the pitch, diameter, and radius, the heat transfer decreases and the change of pressure drop is different. The curvature parameters are introduced in the *Nu* correlation in the case of supercritical CO_2_ cooling heat transfer with an octagon cross section.
Zeinali and Neshat^22^	Turbulent flow, numerical, water	Shell and spirally tube	Temperature difference ratio, and mass flow ratio	The exergetic performance is more sensitive to the variation in the inlet temperature of the shell side rather than to the inlet temperature of the tube side. By increasing the mass flow ratio, the irreversibility rate decreases while the exergy efficiency increases. The PEC value is affected mainly by *R*m compared to *R*T.
Talebi and Lalgani^23^	Turbulent flow, numerical, water	Elliptical spiral tube	Different torsion step-pitch step, three bladed spiral tube-triple turbinate tube, and elliptical spiral tube oval turbinate tube-elliptical helical tube	For the elliptic and triangular spiral tube with a constant and variable torsion step the heat transfer coefficient is higher than the straight tube by 18%, 29%, and 63%, respectively, while the increase in *ΔP* is 65%, 107%, and 140% respectively.
Kumar et al.^24^	Turbulent flow, numerical water	Micro-fin helical tubes	Different coil diameter from 100:200 mm, coil pitch of 25:45 mm and, *Re* varying from 10,000:25000	*Nu* and *ΔP* increase with an increase of fin number and *Re*. Compared to the smooth helical tube, the micro-fin helical tube having a fin number of 12 provides higher *Nu* and *ΔP* in the percentage of 39–51% and 22–36%, respectively. The coil pitches of the helical tube have a small effect on *Nu*. At the same *Re* = 20,000, *Nu* of the smooth helical coiled tube with coil pitch = 25 mm is about 0.86% and 1.71% higher than that of the tube with coil pitch = 35 mm and 45 mm, respectively. Moreover, the performance factor of the micro-fin helical tube with 8 fin declines from 1.08 to 0.96 with an increase of the coil diameter from 100 mm to 200 mm, respectively.
Luo and Song^25^	Turbulent flow, numerical water	Double-tube with twisted annulus	Different aspect ratios and different twist ratios with opposite twisting directions	The maximum *Nu* and *f* of the twisted annuli are separately 157% and 118% larger than those of the corresponding straight annuli. The largest value of the thermal performance factor is 1.98. Correlations of *Nu* and *f* as a function of *Re*, twist ratio, and aspect ratio are proposed.
Naphon et al.^26^	Turbulent flow, numerical, Eulerian two-phase	Spiral tube with/without helical ribs	Different nanofluid concentrations of 0.025%, and 0.050% with and without helical ribs	*Nu* from the spirally coiled tube with helical ribs is higher than by 12.45% compared to the spirally coiled tube without helical ribs. At 0.05% nanofluid concentration, the *Nu* enhanced by 8.21%.
Pethkool et al.^27^	Turbulent flow, experimental, water	Helically corrugated tube	Different pitch ratio (0.18, to 0.27) and different rib-height ratio (0.02, to 0.06), and *Re* from 5500 to 60,000	the heat transfer increased by 123% and 232%, while the maximum thermal performance reach 2.3 for using the corrugated tube with the pitch-to-diameter ratio of 0.27 and the rib-height to diameter ratio of 0.06 at a low *Re*. Correlations of *Nu*,* f*, and thermal performance factor are determined.
Vicente et al.^28^	Turbulent flow, experimental, water and ethylene glycol	Spirally corrugated tubes	Different rib height to diameter ranging from 0.02 to 0.06 and differentspiral pitch to diameter from 0.6 to 1.2., and *Re* from 2000 to 90,000 and *Pr* from 2.5 to 100	A unique dimensionless parameter named severity can be used to establish roughness influence on flow.
Abdelmagied^29^	Turbulent flow, numerical water and Al_2_O_3_/water	Double spiral tube	Different curvature ratios and different Al_2_O_3_/water nanofluid concentrations	Increasing the curvature ratio and nanofluid concentration enhance the heat transfer characteristics at the expense of increasing the pressure drop. Increasing the nanofluid concentration improves *k*.
Alhendal et al.^30^	Turbulent flow, numerical and experimental, water and Al_2_O_3_/water	Double helical tube	Different curvature ratios and different Al_2_O_3_/water nanofluid concentrations	Increasing the curvature ratio enhances the heat transfer characteristics at the expense of increasing the pressure drop, increasing the nanofluid concentrations improves the heat transfer.
Cao et al.^31^	Turbulent flow, numerical and experimental, wat-er and Air	Double helical tube	Different configurations of inner and outur tubes	The outer and inner corrugating tubes provides 125% higher in *Nu* compared to the smooth tubes. The relative position of the corrugated outer and inner tube obtained 35% higher in *Nu* is than the corrugating inner tube with a smooth outer tube.
Present study	Turbulent flow, experimental, water	Twisted tube helical coil	Different inner tube geometry, twisted pitch and coil orientation	Under investigation

Wang et al.^14^ numerically and experimentally studied the thermohydraulic performance of a twisted trilobal helical tube. The results showed that due to the more intense secondary flow of the twisted trilobal helical tube caused by the superposition of overall twist and self-twist, the region near the center of the heat exchanger had higher turbulent kinetic energy, and the region near the wall of the design had a better field synergy angle. Chang et al.^15^ numerically described the turbulent flow and heat transfer of helical coils with twisted sections. The results showed that swirl enhanced *Nu* and *f* for the twisted helical tube relative to the untwisted coil. Yu et al.^16^ demonstrated the turbulent flow thermal-hydraulic performance of twisted oval tubes with different cross-sectioned wire coils, including circular, equilateral, and square cross-sectioned wire coils. A novel hybrid smooth and spiral corrugated tube design was proposed by Yang et al.^17^ with a six-start spirally corrugated part. This study investigated the effects of various structural parameters on thermal and hydraulic performance. Correlations between *Nu* and f as a function of *Re*, the twist ratio, and the aspect ratio are proposed. The thermohydraulic performance of multistarting spiral corrugated tubes was presented by Qian et al.^18^.

Aliabadi and Feizabadi^19^ presented the laminar thermofluidic characteristics of a tubular design by employing a combination of twisted tape and a twisted tube. Reddy et al.^20^ achieved heat transfer and fluid flow at various configurations of the inner tube in a helical tube-in-tube using CFD codes. Yang et al.^21^ employed the RNG k-ε turbulence model with an enhanced wall treatment to determine the influence of supercritical CO_2_ on the heat transfer and pressure drop in a noncircular cross section of a horizontal helical tube. Zeinali and Neshat^22^ analyzed the energy and exergy economics of a shell and spiral tube heat exchanger. The realizable *k*-*ε* turbulence model was employed to evaluate the irreversibility of the reaction. Talebi and Lalgani^23^ investigated the thermal performance of three different models, namely, a novel torsion step-pitch step, a three-bladed spiral tube-triple turbinate tube, and an elliptical spiral tube-oval turbinate tube-elliptical helical tube. Kumar et al.^24^ numerically investigated the characteristics of heat transfer and pressure drop in microfin helically coiled tubes. The thermal performance of a tube-tube heat exchanger with a novel twisted annulus was numerically investigated by Luo and Song^25^. The fluid flow and temperature behaviors of nanofluid flows in spiral tubes with helical ribs were presented by Naphon et al.^26^. Pethkool et al.^27^ investigated heat transfer in turbulent flow by using a helical corrugated tube. Vicente et al.^28^ experimentally investigated the heat transfer and frictional characteristics of spirally corrugated tubes in turbulent flow at different Prandtl numbers. Abdelmagied^29^ and Alhendal et al.^30^ studied the thermal and hydraulic characteristics of different combined techniques of spiral and helical tube heat exchangers with nanofluids. Cao et al.^31^ presented an experimental and numerical study to determine the effect of different structural corrugations on the heat transfer of a helical double-tube heat exchanger. Magar et al.^32^ analysis experimentally the heat transfer of different helicoidal tubes with various curvature ratios. Various coils geometry and curvature ratious were examined. Diwaker and Kumar^33^ derive a study to view the impact of a semi-circular cut twisted tape insert on thermo-hydraulic performance. The study carried out at various twist ratios and cut diameters. The use of the twist and the diameter of the cut increases both the heat transfer and the *Nu*. Abdullah et al.^34^ showed a review of utilizing nanofluids in a helical coil heat exchanger on the thermal performance. The High efficiency of heat exchangers can benefit from several techniques for enhancing their thermal performance.

A few studies have investigated the thermofluid performance of a *TTHC*. Therefore, the present study aims to experimentally investigate the thermofluid characteristics of a *TTHC* at different configurations of the inner tube, including triangular and square profiles. Different *TTHC* tube pitches, $\Upsilon$, of 36 mm and 54 mm, ∞ (smooth/no twisted), are designed, manufactured, and examined.

## Twisted tubes

**Fig. 1:**
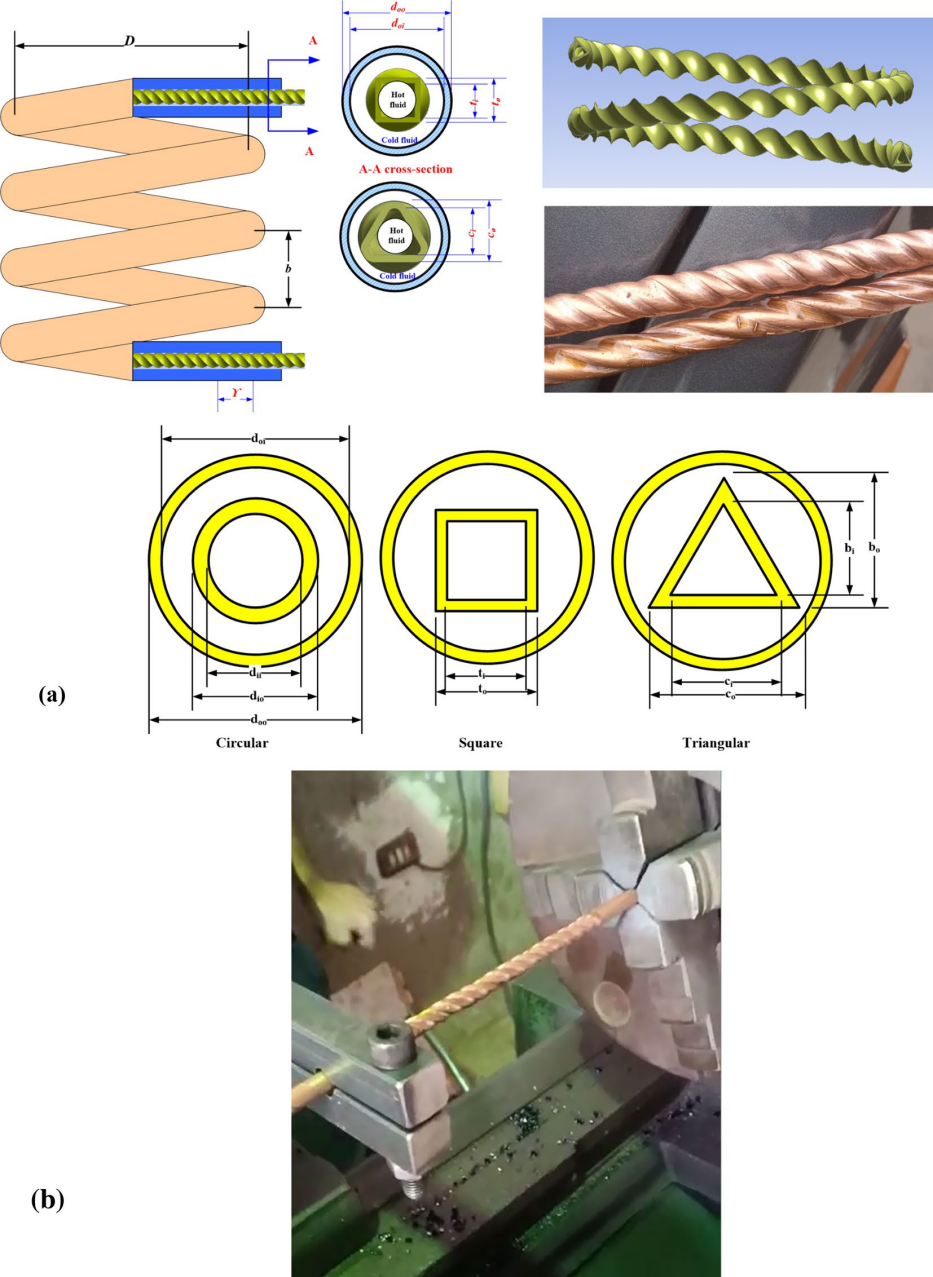
(**a**) Geometries and dimensions of *THTT* inner tubes cross-section profiles, and (**b**) forming process on a lathe.

**Fig. 2:**
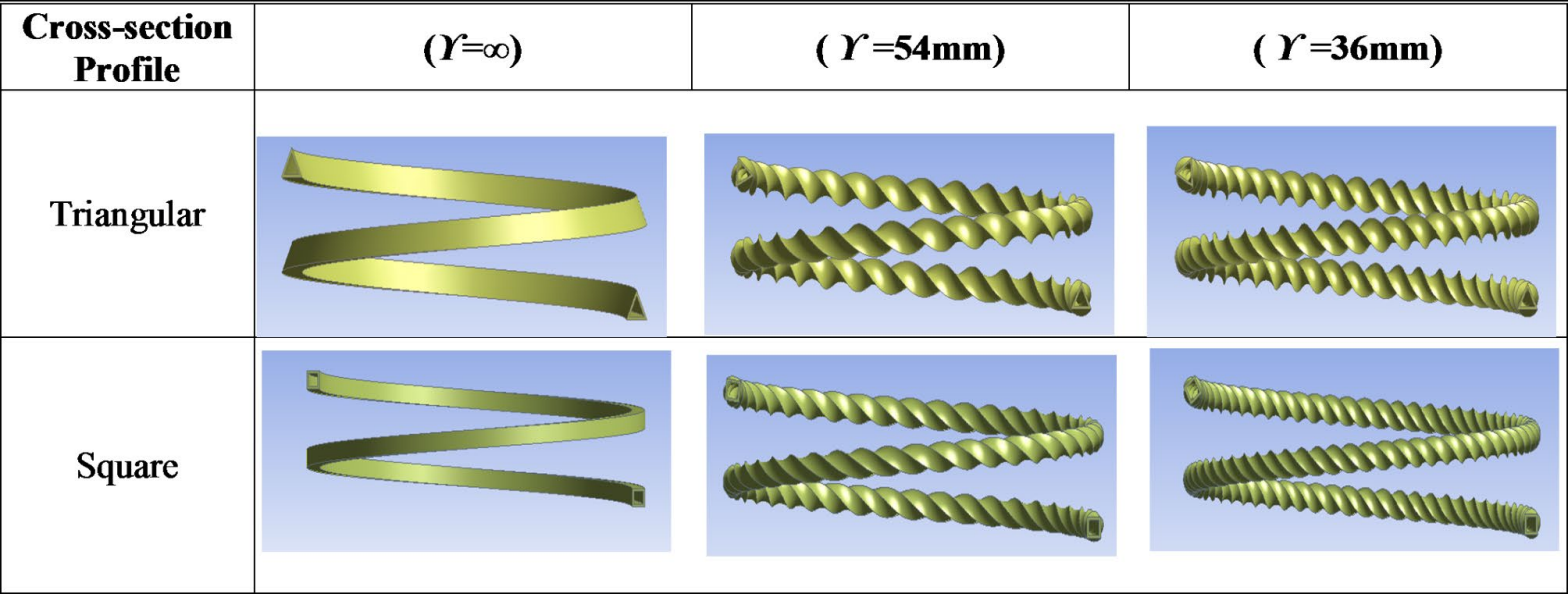
Design profiles of *TTHC* inner tube.

The cross-sectional shape of the test specimens that is expected to enhance the heat transfer characteristics is called a twisted tube and is depicted in Fig. 1a. The twisted tubes were fabricated by forming a normal circular copper straight tube. The end of a circular tube is tagged on the lathe, and the tube is formed by cold drawing using a rotating die on the outer surface, which produces internal twisted grooves similar to the external groove (Fig. 1b). All the tubes had a length of 2 m. The inner diameter and outer diameter of the smooth circular tube before forming are 5.9 mm and 7.9 mm, respectively. After the forming process, the circular tube profile changes to either a triangular or square shape. Six test specimens were examined in the present study with different inner twisted tube pitches of 36 mm, 54 mm, and ∞ with triangular and square cross-sectional profiles (Fig. 2).

## Experimental test rig

**Fig. 3:**
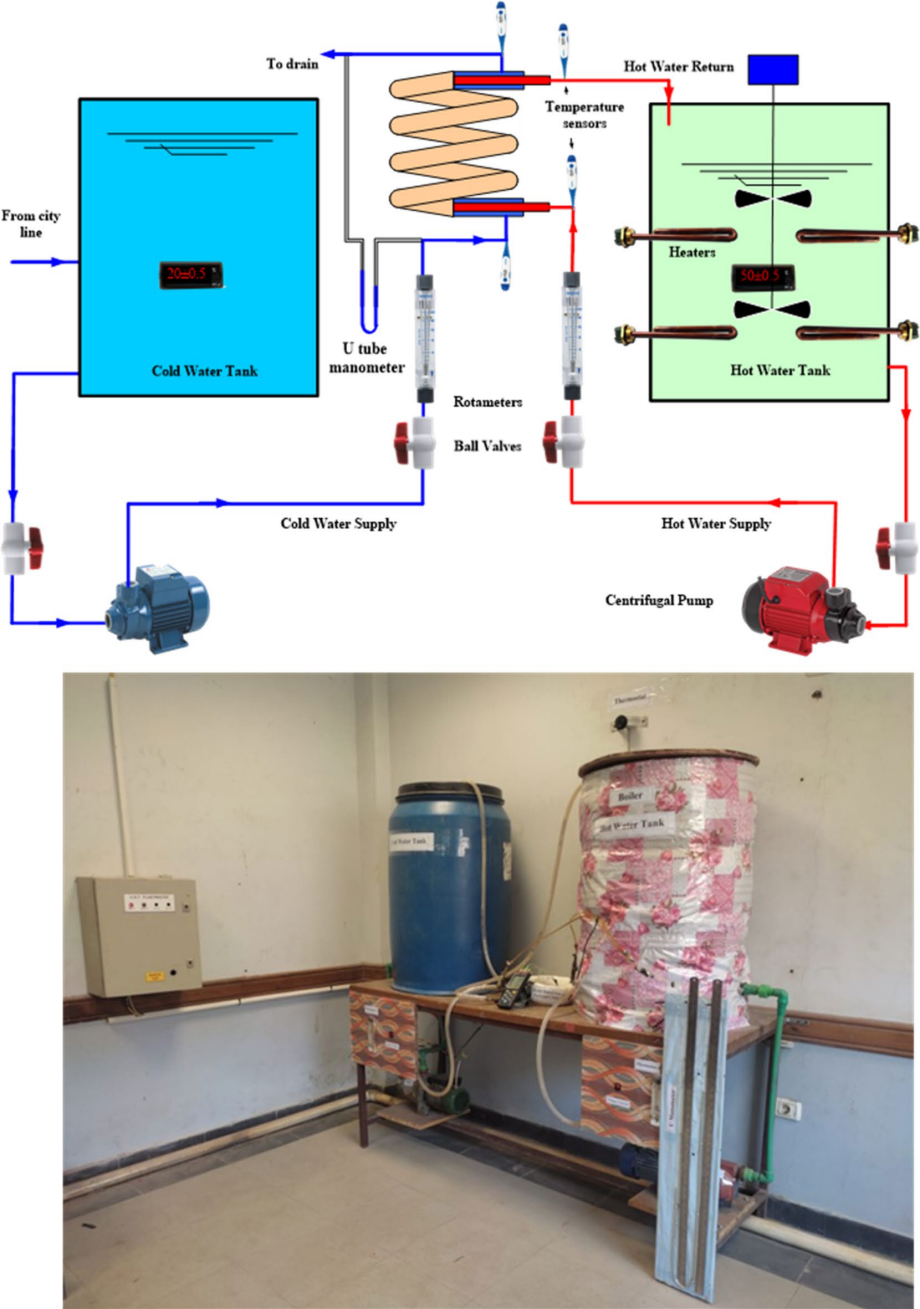
the experimental the test rig. (**a**) schematic diagram and (**b**) photographic picture.

An experimental test rig (Fig. 3) is designed, manufactured, and examined to explore the twisted helical tube-in-tube (*TTHC*) thermofluid characteristics. The test rig consisted of an open water-cooling circuit, a closed hot water circuit, and a *TTHC* circuit. The open-circuit water cooling components include a city water line, an insulated tank of 0.15 m^3^, and temperature adjustment control. The closed hot water circuit components are an insulated 0.2 m^3^ tank, a temperature control adjustable device, and a 6 kW total capacity electric heater. The *TTHC* loop consists of two copper tubes (*ρ* = 8978 kg/m^3^, *C* = 381 J/kg). K, and *k* = 387.6 W/m.K). The inner tube is designed to be a twisted tube with different twisted pitch ratios and cross-sectional profiles. The coil is helically wrapped at a constant diameter, where *R* and *p* are the *TTHC* coil radius and pitch, respectively. The geometrical dimensions are summarized in Table 2. The hot water was fed to the *TTHC* using a 1 h centrifugal pump through the inner tube, and the flow rate was determined by a ball valve and a rotameter from 0.016 to 0.3 kg/s. The hot water was supplied at a temperature of 50 °C ± 0.5 °C. While cold water is pumped at (20 °C ± 0.5 °C) in the *TTHC* outer tube annulus, using a centrifugal pump with a capacity of 1 hp, the flow rates are determined by ball valves and a rotameter (0.016–0.3 kg/s). The outer surface area of the *TTHC* is thermally insulated to decrease heat loss. The temperatures of the *TTHC* inlet and outlet fluids are measured using four precalibrated K-type thermocouples ± 0.1 °C accuracy. The pressure drop across the *TTHC* was measured by a mercury U-tube manometer with (± 1 mm hg accuracy).

**Table 2:** Geometry parameters of *TTHC* (dimensions in mm)

Tube profile	Parameter	Value	Parameter	Value
Outer tube	Inner diameter, *d*_*oi*_	14.1	Outer diameter, *d*_*oo*_	15.8
Inner circular tube	Inner diameter,*d*_*ii*_	5.93	Outer diameter, *d*_*io*_	7.94
Inner triangular tube	Inner height, *b*_*i*_	6.92	Outer height, *b*_*o*_	9.26
	Inner length, *c*_*i*_	7.99	Outer length, *c*_*o*_	10.7
Inner square tube	Inner length, *t*_*i*_	5.3	Outer length, *t*_*o*_	7.03
***-***	Coil diameter, *D*	180	Coil inclination angle	0^o^-45^o^-90^o^
	Coil length, *L*	2000	Coil number of turns, *N*	3.5
	Coil pitch, *b*	31.6	Tube material	Copper
	Inner fluid temperature, °C	50	Annulus fluid temperature, ^o^C	20
	Inner fluid velocity range, m/s	1.2–3.62	Annulus fluid velocity range,	0.3–2.8 m/s

## Data reduction

The thermofluid characteristics of the *TTHC* can be obtained by defining the Reynolds number (*Re*) parameter as follows:

1 $$Re=\frac{{\rho u{d_h}}}{\mu }$$

The heat supplied from the hot fluid and heat absorbed by the cold fluid can be expressed as:

2 $$\mathop {{Q_h}}\limits^{.} =\mathop {{m_h}}\limits^{ \cdot } {C_h}\left( {{T_{h,\,i}} - {T_{h,\,o}}} \right)$$

3 $$\mathop {{Q_c}}\limits^{.} =\mathop {{m_c}}\limits^{ \cdot } {C_c}\left( {{T_{c,\,o}} - {T_{c,\,i}}} \right)$$

The overall heat transfer coefficient can be written as:

4 $$\,{U_o}=\frac{{\mathop {{Q_{Avg.}}}\limits^{.} }}{{\pi {d_{i,o}}L\,\Delta {T_{LMTD}}}}$$

where the average rate of heat transfer can be calculated as

5 $${Q_{Avg.}}=0.5\left( {{Q_c}+{Q_h}} \right)$$

**Fig. 4:**
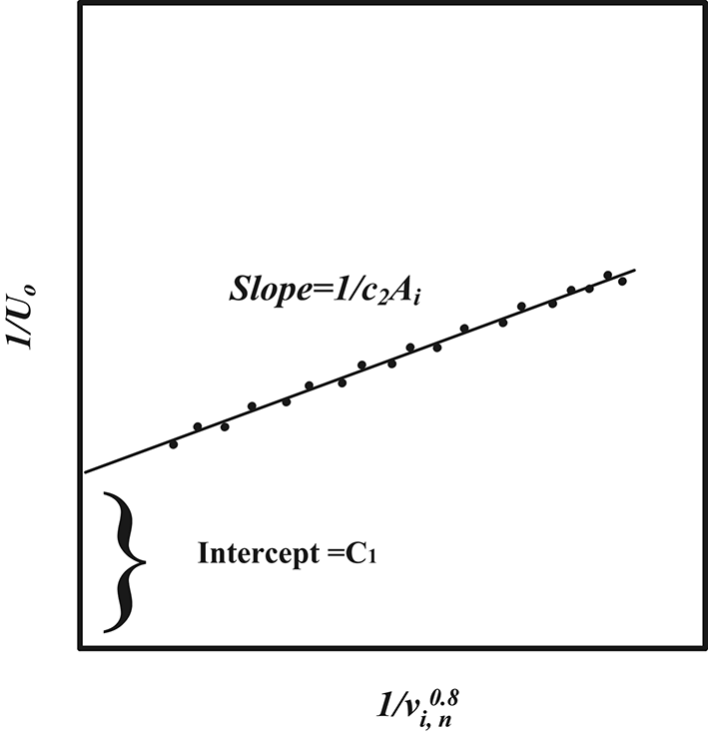
Wilson plot diagram^33^.

According to the traditional Wilson plot method^35,36^, the heat transfer coefficients of the *TTHC* for both the inner and annulus sides can be determined based on the overall temperature difference and the heat transfer rate without the requirement of tube surface temperatures. This method is chosen to avoid disturbance of flow patterns and heat transfer while attempting to measure tube surface temperatures. Wilson plots are generated by calculating the overall heat transfer coefficients for several trials. One fluid flow side is kept constant, and the other fluid flow side is varied, and vice versa (Fig. 4). Where *v*_*i*_ refers to the water velocity on the inner tube side, *A*_*i*_ is the inner tube surface area, and *n* is an exponent that is approximately 0.8. The inner tube side convective heat transfer coefficient, *h*_*i*,_ can be determined as follows:

6 $${h_i}={c_2}v_{i}^{n}$$

By calculating *h*_*i*_, the annulus side convective heat transfer coefficient, *h*_*o*,_ can be determined from the overall heat transfer coefficient relationship as^37^:

7 $$\frac{1}{{\mathop U\nolimits_{o} }}=\frac{{\mathop A\nolimits_{o} }}{{\mathop A\nolimits_{i} \mathop h\nolimits_{i} }}+\frac{{\mathop A\nolimits_{o} \ln \left( {{d_o}/{d_i}} \right)}}{{2\pi kL}}+\frac{1}{{\mathop h\nolimits_{o} }}$$

The Nusselt number is determined as follows:

8 $$\,N{u_o}=\frac{{{h_o}{d_h}}}{{{k_o}}}$$

The hydraulic diameter *d*_*h*_ is the annulus side and can be defined (Table 3) as follows:

9 $${d_h}=\frac{{4{A_{c.\operatorname{s} }}}}{{\,wp}}=\frac{{\pi /4d_{{o,i}}^{2} - {A_{o,\,it}}}}{{\pi {d_{o,i}}+w{p_{o,\,it}}}}$$

**Table 3:** Annulus hydraulic diameter calculations of *TTHC*.

Inner tube profile	Para-meter	Value, mm	Annulus cross-section area, A_c.s_, mm^2^		Annulus wetted perimeter, wp, mm	Hydraulic diameter	Annulus d_h_, mm
Outer tube	*d*_*oi*_	14.1	$\frac{\pi }{4}\left( {{d_{{\text{oi}}}}^{{\text{2}}} - d_{{{\text{io}}}}^{{\text{2}}}} \right)$	49.5	$\pi \left( {{d_{oi}}+{d_{io}}} \right)$	$\frac{{4{A_{c.s}}}}{{wp}}$	6.2
Circular	*d*_*io*_	7.94					
Triangular	*b*_*o*_	9.26	$\left( {\frac{\pi }{4}{\text{ }}{d_{{\text{oi}}}}^{{\text{2}}}} \right) - 0.5\,{b_o}\,{c_o}$	49.5	$\pi {d_{oi}}+{\text{3}}{c_o}$		5.6
	*c*_*o*_	10.7					
Square	*t*_*o*_	7.03	$\left( {\frac{\pi }{4}{\text{ }}{d_{{\text{oi}}}}^{{\text{2}}}} \right) - t_{{_{o}}}^{2}$	49.5	$\pi {d_{oi}}+{\text{4}}{t_o}$		7

The Darcy friction factor is calculated as follows:

10 $${f_o}=\frac{{2\Delta {P_o}}}{L}.\frac{{{d_h}}}{{\rho {v_o}^{2}}}$$

The exergy balance equation for a *TTHC* can be expressed as follows^38^:

11 $$\mathop {{E_{in}}}\limits^{.} =\mathop {{E_{out}}}\limits^{.} +\mathop {{E_{loss}}}\limits^{.}$$

12 $$\mathop {{E_{in}}}\limits^{.} ={m_c}\left[ {Cp\left( {{T_{c,i}} - {T_0}} \right) - Cp{T_0}Ln\left( {\frac{{{T_{c,i}}}}{{{T_0}}}} \right)+\frac{{v_{c}^{2}}}{2}} \right]+{m_h}\left[ {Cp\left( {{T_{h,i}} - {T_0}} \right) - Cp{T_0}Ln\left( {\frac{{{T_{h,i}}}}{{{T_0}}}} \right)+\frac{{v_{h}^{2}}}{2}} \right]$$

13 $$\mathop {{E_{out}}}\limits^{.} ={m_c}\left[ {Cp\left( {{T_{c,o}} - {T_0}} \right) - Cp{T_0}Ln\left( {\frac{{{T_{c,o}}}}{{{T_0}}}} \right)+\frac{{v_{c}^{2}}}{2}} \right]+{m_h}\left[ {Cp\left( {{T_{h,o}} - {T_0}} \right) - Cp{T_0}Ln\left( {\frac{{{T_{h,o}}}}{{{T_0}}}} \right)+\frac{{v_{h}^{2}}}{2}} \right]$$

Hence, the exergy efficiency can be defined as follows:

14 $${\eta _{Ex.}}=\frac{{\mathop {{E_{out}}}\limits^{.} }}{{\mathop {{E_{in}}}\limits^{.} }}$$

The exergy destruction rate (exergy loss) can be calculated as follows:

15 $$\mathop I\limits^{.} =\mathop {{E_{in}}}\limits^{.} - \mathop {{E_{out}}}\limits^{.}$$

## Measurements uncertainty

The uncertainty of the experimental error should be considered when implementing the error in the results. Holman^39^ determines the error of the measured quantities to compute the uncertainty of several parameters such as *Re*,* h*,* Nu*,* f*, and *U*_*o*_. For the independent variables (*t*_*1*_, *t*_*2*_, *s*_*3*_, …, *t*_*n*_), considering the uncertainty in *Y*_*1*_, *Y*_*2*_, …, *Y*_*n*_, and *Y*_*R*_, the uncertainty in the experimental result was of the same order of magnitude, which can be given as follows;

16 $${Y_R}=\sqrt {{{\left( {\frac{{\partial R}}{{\partial {t_1}}}{Y_1}} \right)}^2}+{{\left( {\frac{{\partial R}}{{\partial {t_2}}}{Y_2}} \right)}^2}+\,\,.\,\,+{{\left( {\frac{{\partial R}}{{\partial {t_n}}}{Y_n}} \right)}^2}}$$

The results of measuring uncertainty are given in Table 4.

**Table 4:** The range, accuracy and uncertainties of measuring devices.

Instruments/parameter	Range	Accuracy (%)	Uncertainty (%)
Rotameter, kg/s	0.016–0.3	± 0.5	2.7
Thermocouple K-type, °C	-200-1200	± 0.1	0.2
U tube manometer, mm Hg	0.5–500	± 1	± 0.13
*Re*	37,000 − 3000	-	± 2.87
Friction factor	-	-	± 3.1
*U*_*o*_, W.m^− 2^ °C^−1^	-	-	± 6.79
*h*_*o*_, W.m^− 2^ °C^−1^	-	-	± 3.27
*Nu*	-	-	± 3.29

## Results and discussions

### Verification of smooth and twisted tubes

**Fig. 5:**
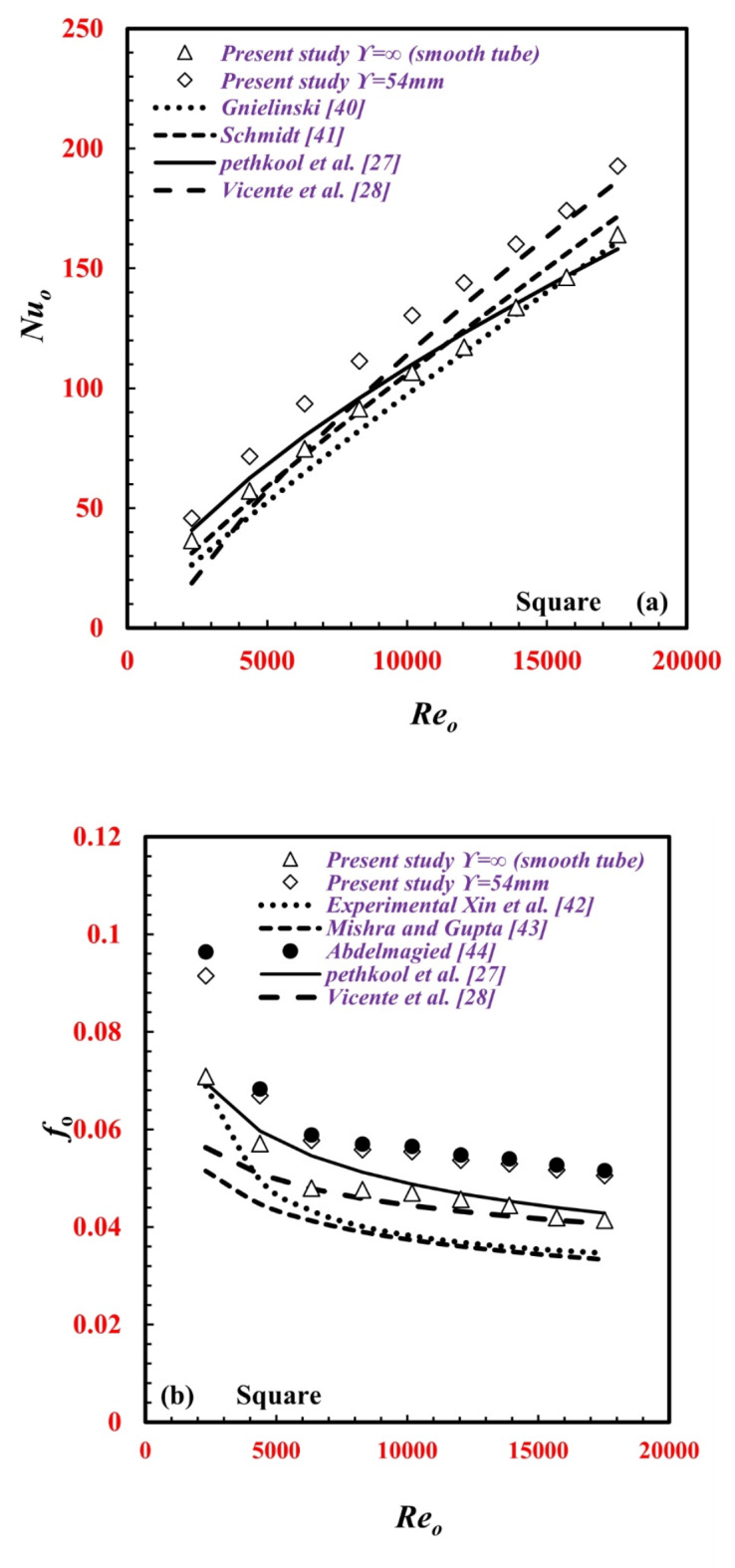
Validation between the present versus and the previous studies, (**a**) *Nu*_*o*_ abd (**b**) *f*_*o*_.

First, the present results for the smooth tube and twisted tube of the experimental facility are validated by comparing the heat transfer rate in terms of *Nu* with those from published correlations, including the Genliniski^40^ correlation for *Nu* and Schmidt^41^ for smooth tubes and the correlations of Pethkool et al.^27^ and Vicente et al.^28^ for twisted tubes, as depicted in Fig. 5a. The present data for the smooth tube agree well with the correlations of Genliniski^40^ and Schmidt^41^ by ± 13.1% for ± 4.8%, while the present results for the twisted tube also agree well with the correlations of Pethkool et al.^27^ and Vicente et al.^28^ by ± 17% for and ± 6.9%, respectively.

The pressure drop behaviors in terms of *f* are also compared with the correlations of Xin et al.^42^ Mishra and Gupta^43^, and Abdelmagied^44^ for a smooth tube and Pethkool et al.^27^ and Vicente et al.^28^ for a twisted tube, as depicted in Fig. 5b. The present data for smooth tubes agree well with those from the correlations of Xin et al.^42^, Mishra and Gupta^43^ and Abdelmagied^44^ by ± 23.9%, ± 26.8%, and ± 2%, respectively. The present results also agree well with the correlations of Pethkool et al.^27^ and Vicente et al.^28^ for twisted tubes by ± 14.5% and ± 18%, respectively.

### Thermal analysis

**Fig. 6:**
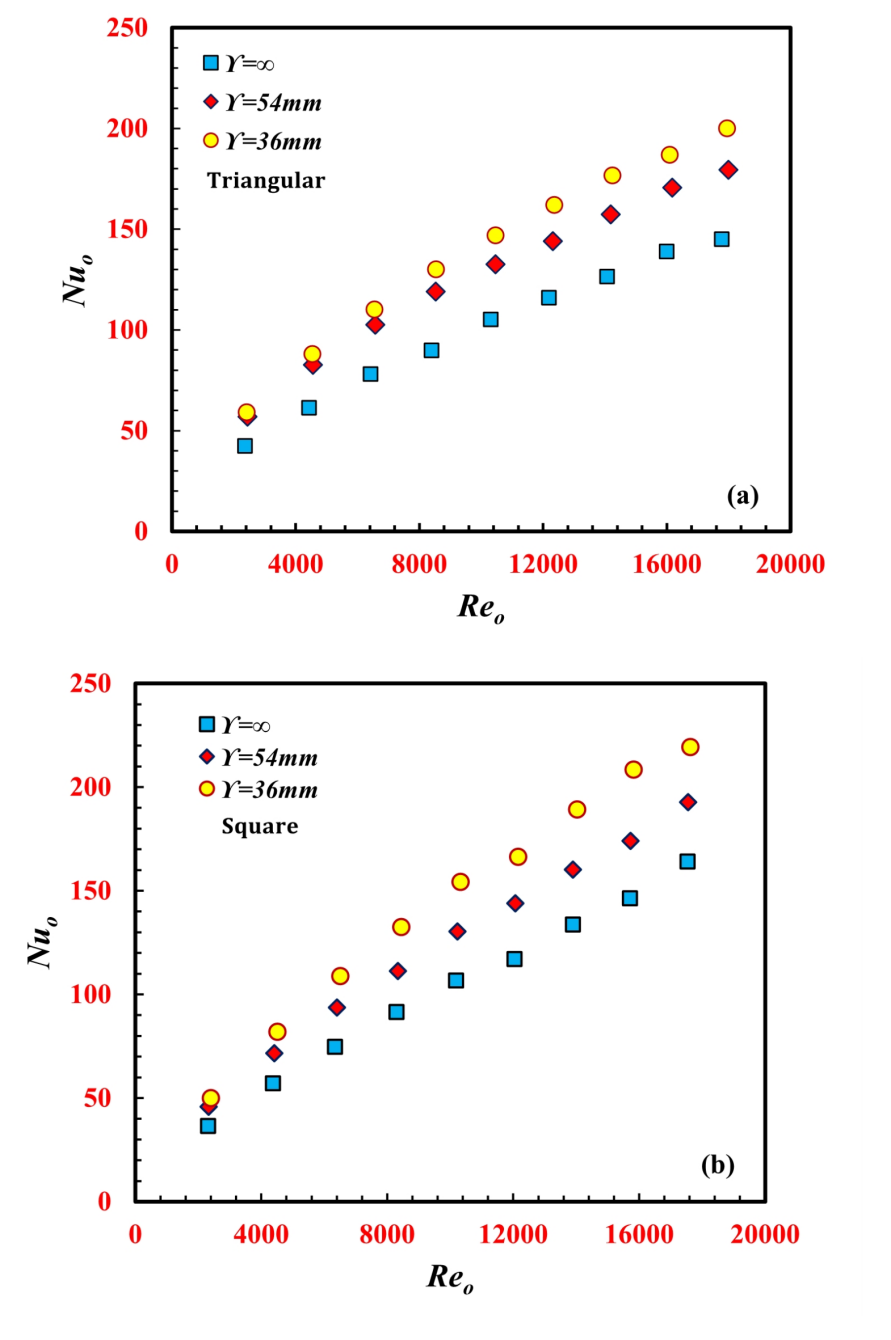
Variation of *Nu*_*o*_ against *Re*_*o*_ for for different *TTHC* inner twisted tube (**a**) Triangular and (**b**) Square.

Figure 6a and b plots the annulus *Nu*_*o*_ versus *Re*_*o*_ for both the *TTHC* triangular and the square inner tube designs and for three twisted pitches, $\Upsilon$, of 36 mm, 54 mm, and *∞* (not twisted). For all the cases, it can be noted from the figure that *Nu*_*o*_ increases with *Re*_*o*_ for all the cases and is greater than the number of smooth tubes. This can be referred to as an increase in the fluid velocity and turbulence level, in addition to enhancing the secondary flow that is generated due to the centrifugal force that is created by fluid flow in a curved tube. This leads to an increase in the convective heat transfer coefficient and an increase in the heat transfer rate. As shown in the figure, with decreasing twisted pitch, the twisted tube increases *Nu*_*o*_ through the *TTHC* of the inner tube by increasing the flow resistance and fluid turbulence intensity through the thermal boundary layer. This leads to complete mixing of the thermal boundary layer through the annulus space. By diminishing the thermal boundary layer, the heat transfer rate increased, and Nuo was enhanced. At a particular *Re*_*o*_ of 14,050, the N*u*_*o*_ values for $\Upsilon$ = 36 mm and $\Upsilon$ = 54 mm are 39.6% and 24.3%, respectively, greater than that for $\Upsilon$*=∞* (smooth tube/no twisted) for the triangular inner twisted tube design. Additionally, at a particular *Re*_*o*_ of 14,050, the *Nu*_*o*_ values for $\Upsilon$ = 36 mm and $\Upsilon$ = 54 mm are 41.5% and 19.7% greater than that for $\Upsilon$=∞, respectively, for the square inner twisted tube design. For the same operating conditions, as the number of inner twisted tube edges increases from three edges (triangle shape) to four edges (square shape), the enhancements in *Nu*_*o*_ for $\Upsilon$*=∞*,* 36 mm*, and 54 mm increase by 13.1%, 7.3%, and 9.7%, respectively.

**Fig. 7:**
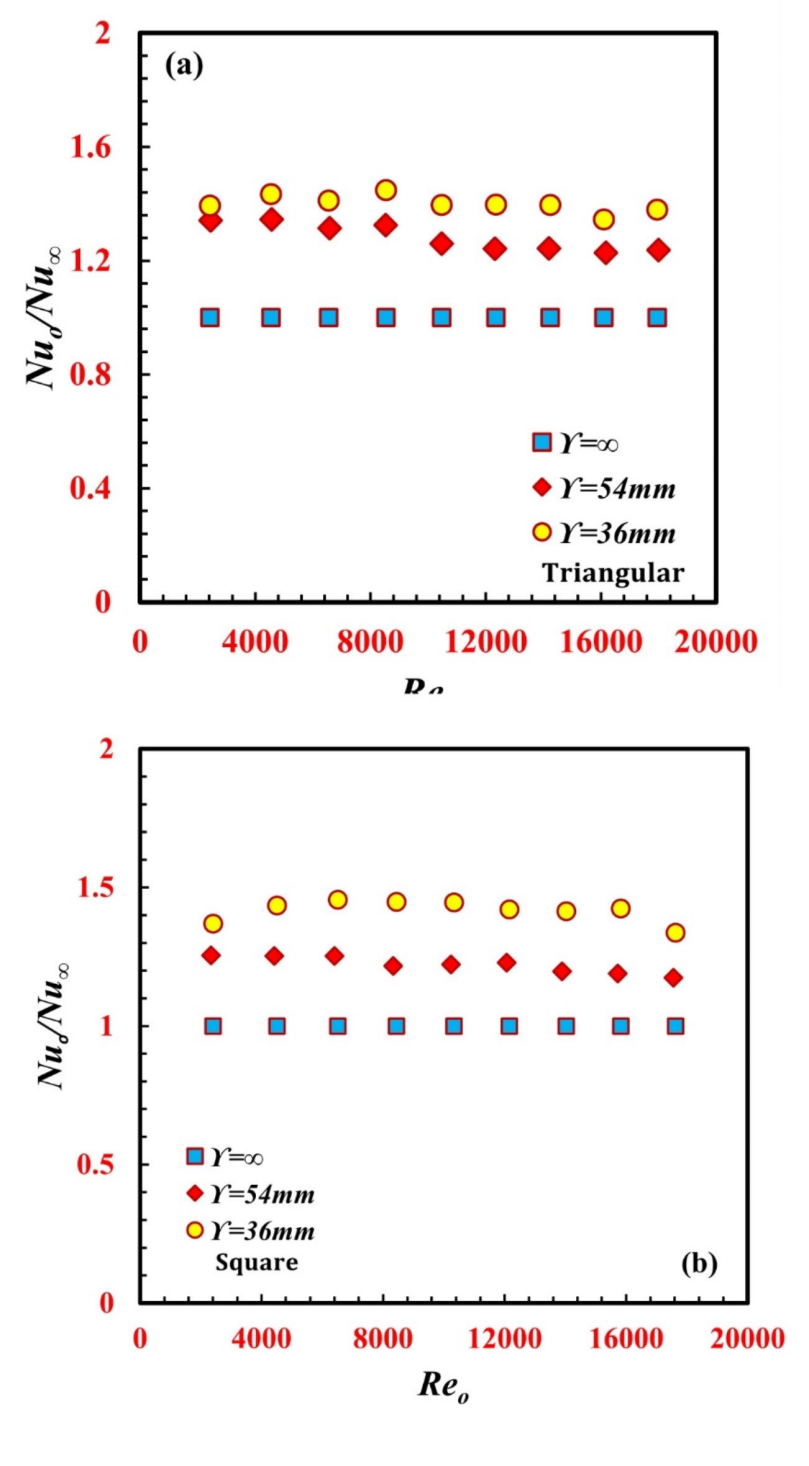
Variation of *Nu*_*o*_*/Nu*_*∞*_ against *Re*_*o*_ for different *TTHC* inner twisted tube (**a**) Triangular and (**b**) Square.

The variation in the ratio between the enhanced and nonenhanced inner twisted tubes on the annulus Nusselt number ratio *Nu/Nu*_*∞*_ is illustrated in Fig. 7(a and b) for both the triangular and square inner twisted tubes of the *TTHC* heat exchanger. It can be shown from the figures that *Nu/Nu*_*∞*_ is reduced with an increase in *Re*_*o*_ and is always greater than unity in all the cases. The maximum enhancement reaches 1.37 for the triangular inner twisted tube at $\Upsilon$ = 36 mm and a*Re* of 8400, while the maximum enhancement reaches 1.46 for the square inner twisted tube at $\Upsilon$ = 36 mm and *a Re* of 6500.

**Fig. 8:**
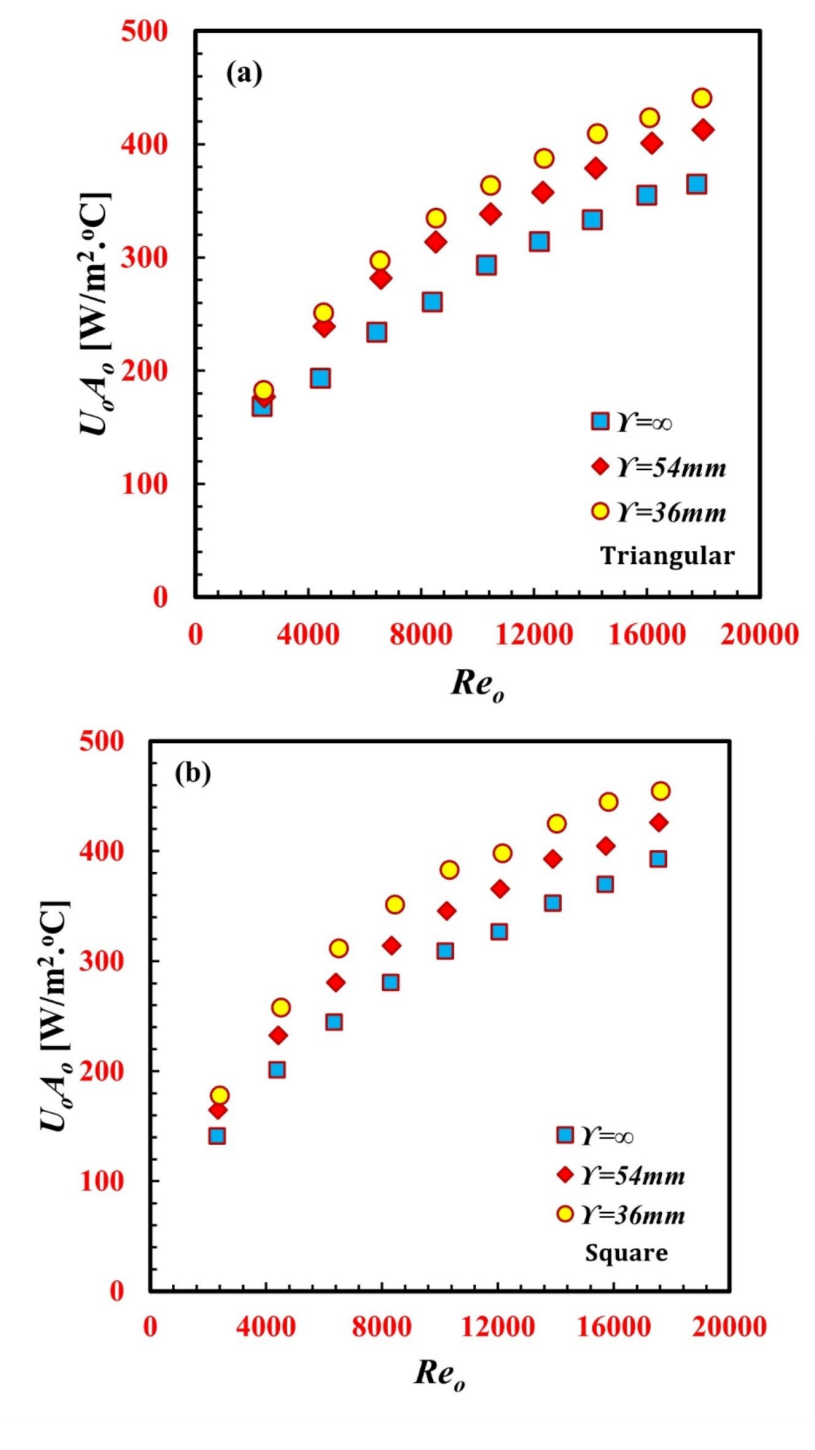
Variation of *U*_*o*_*A*_*o*_ against *Re*_*o*_ for for different *TTHC* inner twisted tube (a) Triangular and (**b**) Square.

Figure 8a, b shows the relationships between the *U*_*o*_*A*_*o*_ product and the *Re*_*o*_ of various twisted pitches and inner twisted tube designs. The figures show that the *U*_*o*_*A*_*o*_ product increased with *Re*_*o*_ in all the cases and was higher than the values for both triangular and square smooth tubes. This can be attributed to the increase in the heat transfer rate due to swirl flow caused by twisted tubes, which increases fluid turbulence, generates secondary flow, and consequently enhances heat transfer characteristics. For a single *Re* of 14,050, the *U*_*o*_*A*_*o*_ products for $\Upsilon$ = 36 mm and $\Upsilon$ = 54 mm are 22.8% and 13.6% greater than that for $\Upsilon$ =∞, respectively, for the triangular inner twisted tube. Additionally, for the single particular *Re* of 14,050, the *U*_*o*_*A*_*o*_ product of $\Upsilon$ = 36 mm is 20.5% and 10.2% greater than that of $\Upsilon$ = 54 mm and $\Upsilon$ =*∞*, respectively, for the square inner twisted tube.

### Frictional analysis

**Fig. 9:**
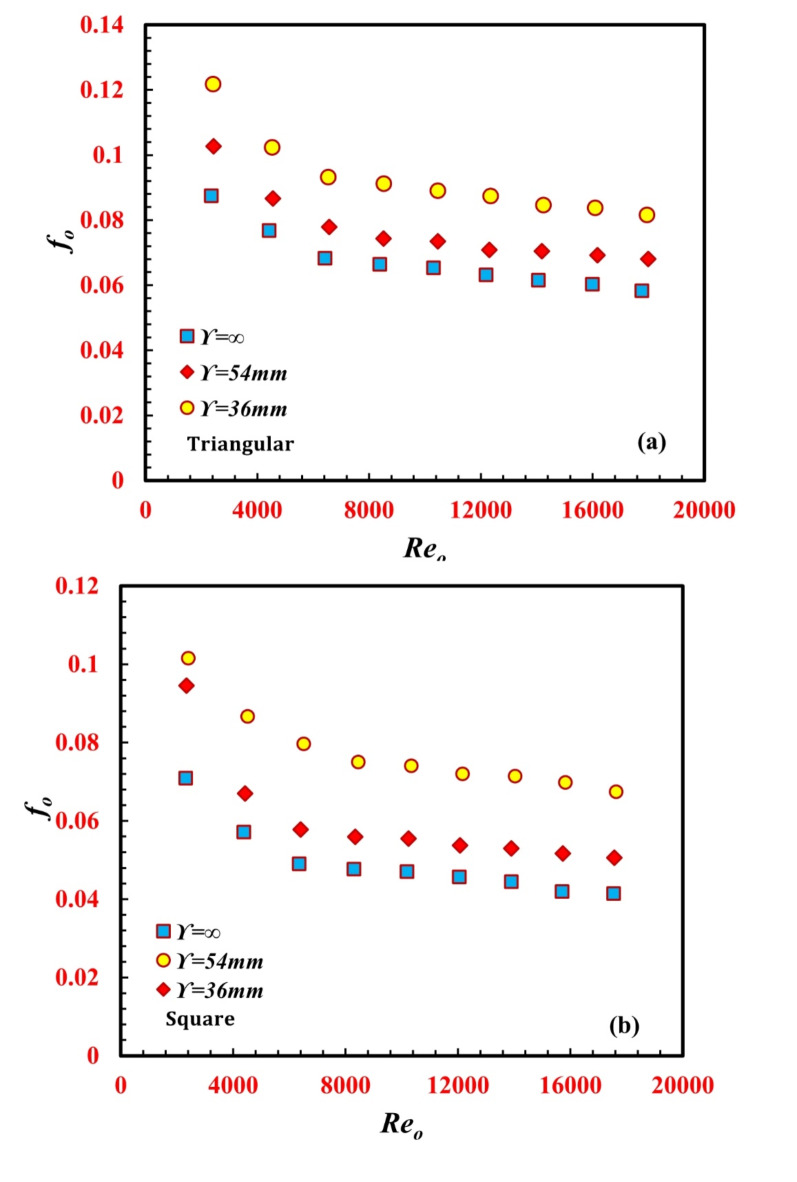
Variation of *f*_*o*_ against *Re*_*o*_ for for different *TTHC* inner twisted tube (**a**) Triangular and (**b**) Square.

**Fig. 10:**
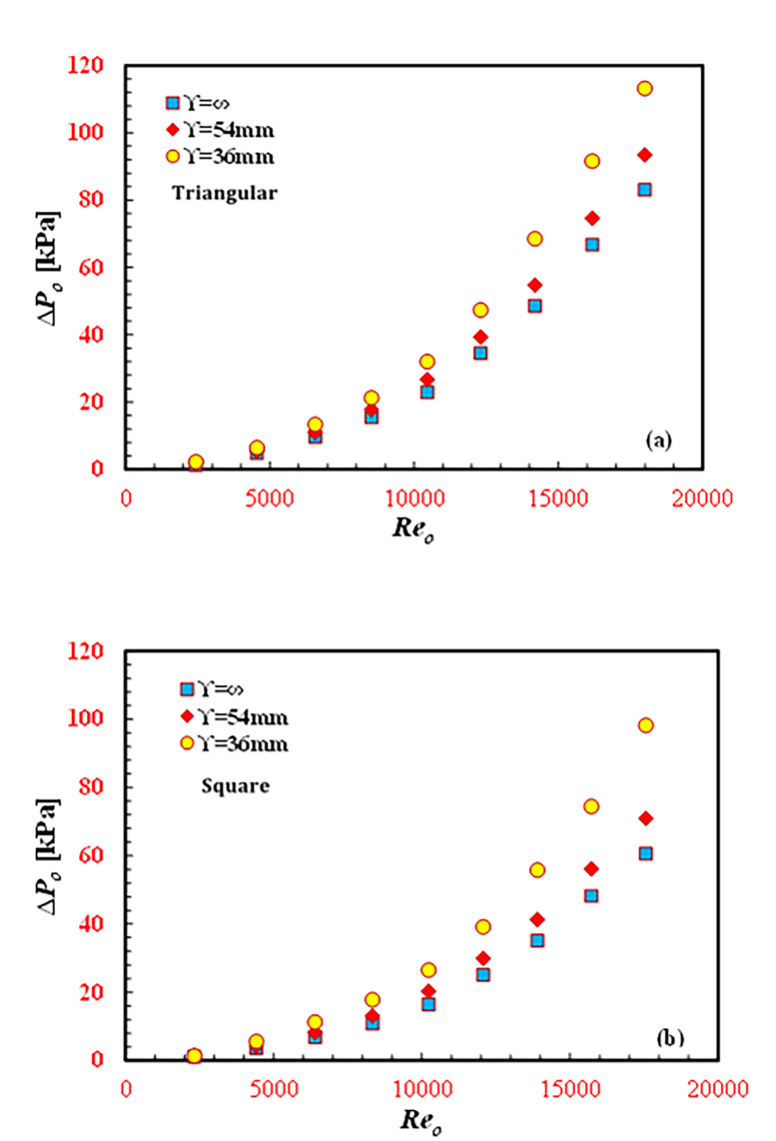
Variation of *∆P*_*o*_ against *Re*_*o*_ for different *TTHC* inner twisted tube (**a**) Triangular and (**b**) Square.

The application of passive techniques to enhance heat transfer resulted in an unavoidable increase in the pressure drop, *∆P*_*o*_. However, the *Nu-*enhanced *∆P*_*o*_ tends to increase as well. Both key parameters, *∆P*_*o*_ and *f*_*o*,_ are demonstrated in Fig. 9(a and b) and 10(a and b). While *∆P*_*o*_ increases with *Re*_*o*_, *f*_*o*_ decreases with increasing *Re*_*o*_. According to Eq. 11, as *Re* increases both the *∆P*_*o*_ and the square of the water velocity, *v*_*o*_^2^ increases. Hence, the behavior of the *f*_*o*_ curve depends on the rate of increase in *∆P*_*o*_ and *v*_*o*_^2^. Therefore, the rate of increase in *v*_*o*_^2^ is greater than that for *∆P*_*o*_. This finding highlights why *f*_*o*_ decreases with increasing *Re*_*o*_. Additionally, by increasing *Re*_*o*,_ the momentum force, centrifugal force, secondary flow, and eddies increase, leading to a decrease in the viscous boundary layer thickness (viscous force) and hence a decrease in *f*_*o*_. By decreasing the twisted pitch, the fluid turbulence, flow resistance and secondary flow increase. An increase in secondary flow tends to increase fluid disturbance, consequently increasing the formation of the generated vortices and hence increasing the annulus friction factor. At a particular *Re*_*o*_ of 14,050, the f values for $\Upsilon$ = 36 mm and $\Upsilon$ = 54 mm are 37.6% and 14.5%, respectively, greater than that for $\Upsilon$*=∞* (smooth tube/no twisted) for the triangular inner twisted tube design. Additionally, at a particular *Re* of 14,050, the *f* values for $\Upsilon$ = 36 mm and $\Upsilon$ = 54 mm are 60.7% and 19.2%, respectively, greater than that for $\Upsilon$=∞ for the square inner twisted tube design. For the same operating conditions, as the number of inner twisted tubes on the edges increased from three edges (triangle shape) to four edges (square shape), the *f*_*o*_ decreased for $\Upsilon$*=∞*,* 6*, and 9 by 40.8%, 34.5%, and 21%, respectively.

**Fig. 11:**
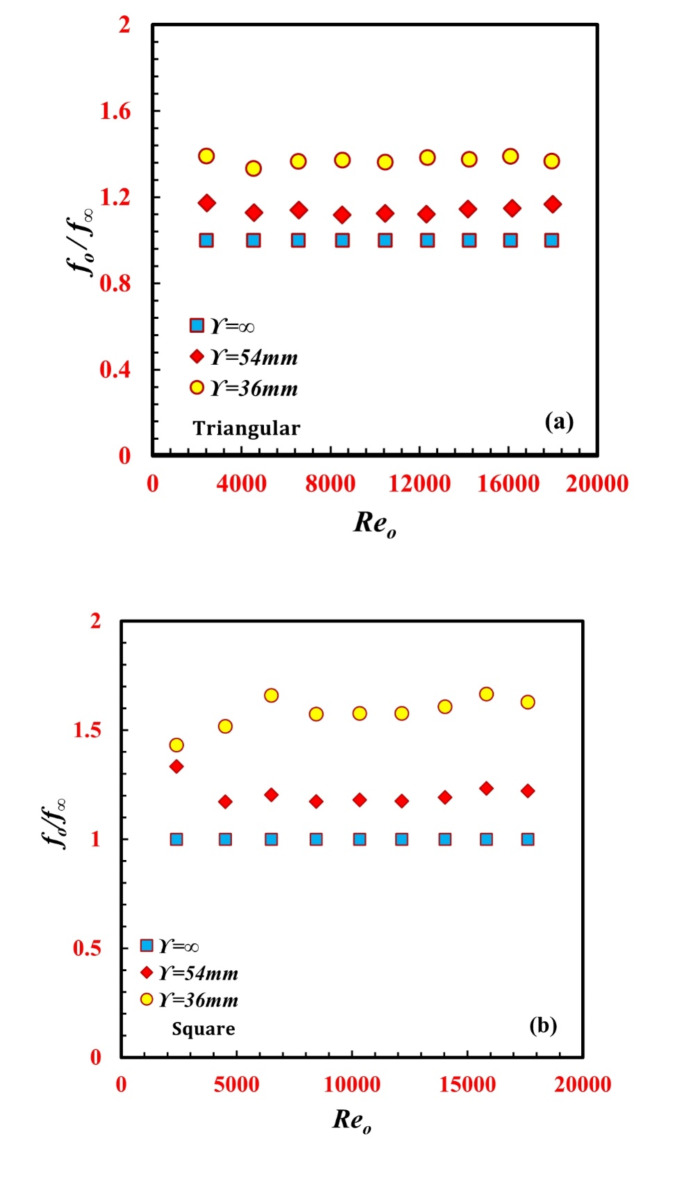
Variation of *f*_*o*_*/f*_*∞*_ against *Re*_*o*_ for different *TTHC* inner twisted tube (**a**) Triangular and (**b**) Square.

The variation in the ratio between the enhanced and nonenhanced inner twisted tubes on the annulus friction factor and *f/f*_*∞*_ is illustrated in Fig. 11(a and b) for both *the* triangular and the square inner twisted tubes of the *TTHC* heat exchanger. It can be noted from the figures that *f/f*_*∞*_ is greater than unity in all the cases. There is a noticeable increase in the *f/f*_*∞*_ in the inner twisted tube helical coil. In all the cases, the maximum *f/f*_*∞*_ value reached 1.39 for the triangular inner twisted tube at $\Upsilon$_*∞*_ = 36 mm and *a Re* of 2400, while the maximum *f/f* value reached 1.63 for the square inner twisted tube at $\Upsilon$ = 36 mm *and a Re* of 6500.

### Thermal performance and exergetic analysis

**Fig. 12:**
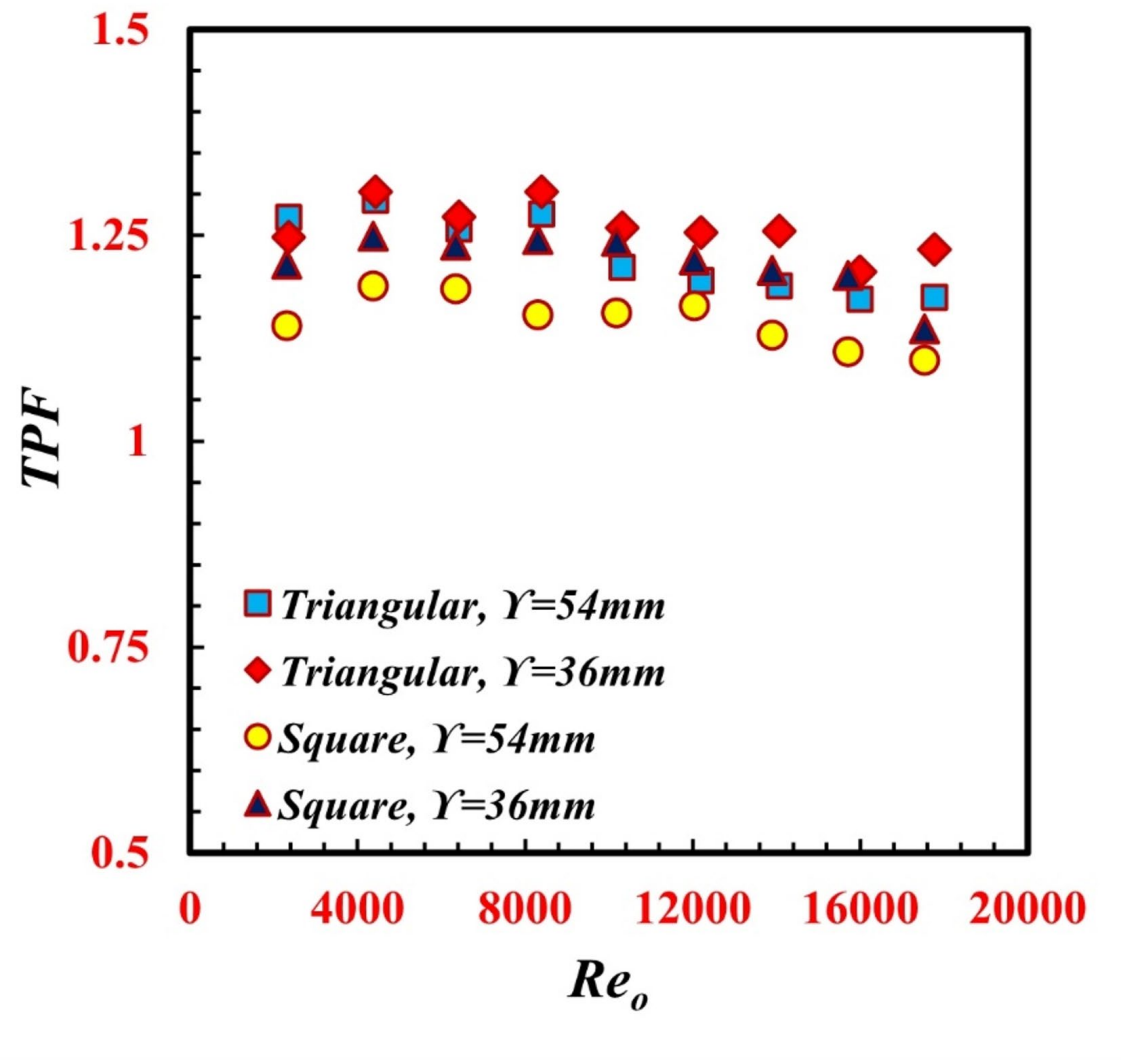
Variation of *TPF* against *Re*_*o*_ for different *TTHC* inner twisted tube.

**Fig. 13:**
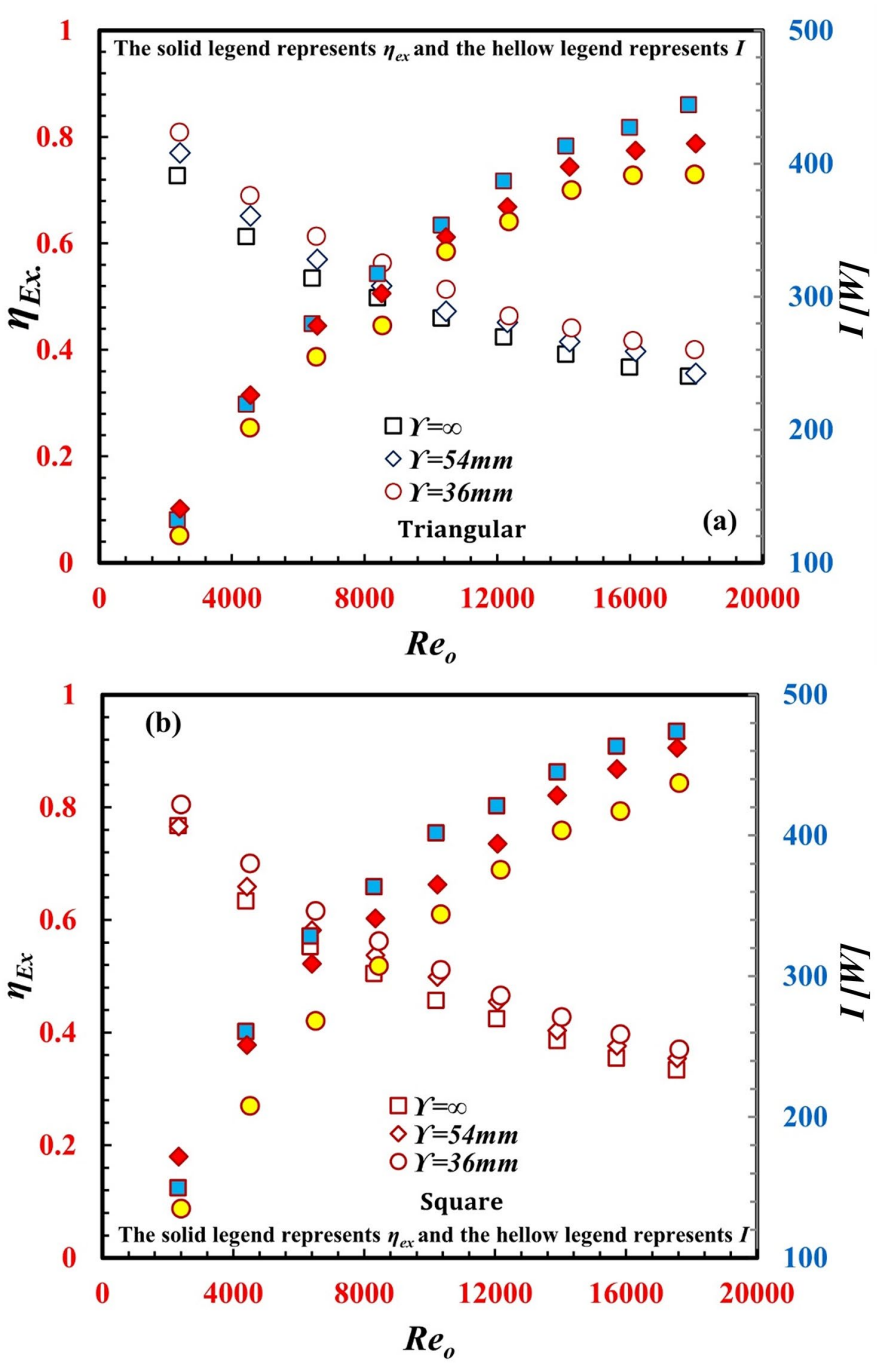
Variation of *η*_*Ex.*_against *Re*_*o*_ for different *TTHC* inner twisted tube (**a**) Triangular and (**b**) Square.

The thermal performance factor (*TPF*) of a *TTHC* is a major point of interest. The *TPF* was used to determine the extent to which heat transfer was augmented, which led to increased pumping power compared to that of smooth tube-in-tube heat exchangers. The *TPF* is the ratio between the augmentations of the Stanton number and the friction factor ratio under the same study flow conditions. Figure 12 represents the *TPF* against *Re* for different $\Upsilon$ and various inner tube designs of the *TTHC* in the counterflow pattern. The *TPF* decreased with increasing Reo. In addition, the minimum *TPF* value is greater than unity. The maximum TPFs reached 1.3 and 1.25 for a $\Upsilon$ of 36 mm for triangular and square inner twisted tube shapes, respectively. The behavior of the *TTHC* can also be evaluated from the viewpoint of the second law of thermodynamics. Exergy analysis, which is a key parameter in the whole system’s economic evaluation, is the main point of interest in this study. By considering the *TTHC* system as a smaller subset, in such systems, the whole system’s exergoeconomic characteristics are evaluated, considering the exergoeconomic characteristics of any subset of equipment. Through the *TTHC*, the exergy loss, *I*, and exergy efficiency, *η*_*Ex.*_, are demonstrated in Fig. 13a and b. The twist of the inner tube in the *TTHC* causes further exergy loss. Generally, any augmentation technique of the heat transfer method causes a heat transfer rate. The heat transfer rate due to temperature differences is one of the key factors in irreversibility and exergy destruction. Thus, exergy destruction because of passive techniques is unavoidable due to the enhanced heat transfer rate. Additionally, as *Re*_*o*_ increased, *I* also increased for the same reason. However, with increasing *Re*_*o*,_*η*_*Ex.*_ is reduced. From the viewpoint of the second law of thermodynamics, the *TTHC* should be applied for lower *Re*_*o*_ values to obtain relatively higher *η*_*Ex values.*_ Cao et al.^31^. The *I* values at $\Upsilon$ = 36 mm and $\Upsilon$ = 54 mm are lower than those at ∞ by 8% and 3.8%, respectively, for the triangular inner twisted tube. Additionally, the *I* values at $\Upsilon$ = 36 mm and $\Upsilon$ = 54 mm are lower than those at ∞ by 9.4% and 3.7%, respectively, for the square inner twisted tube. This represents why $\Upsilon$ = 36 mm provides a higher *η*_*Ex.*_ compared to $\Upsilon$ = 54 mm and ∞.

### Data correlations

As a significant effect of the twisted pitch on the *TTHC* thermal performance, the following correlations predict *Nu*_*o*_ (Eqs. 17 and 19) and *f*_*o*_ (Eqs. 18 and 20) as a function of *Re*_*o*_ and *Pr*_*o*_. A regression method of data analysis was employed as follows:

For the inner twisted triangular tube:

17 $${{N{u_o}} \mathord{\left/ {\vphantom {{N{u_o}} {N{u_\infty }}}} \right. \kern-0pt} {N{u_\infty }}}={\text{2}}{\text{.96}}Re_{o}^{{{\text{0.0217}}}}P{r_o}^{{{\text{-0.583}}}}$$

18 $${{{f_o}} \mathord{\left/ {\vphantom {{{f_o}} {{f_\infty }}}} \right. \kern-0pt} {{f_\infty }}}={\text{2}}{\text{.041}}Re_{o}^{{{\text{-0.05}}}}$$

**Fig. 14:**
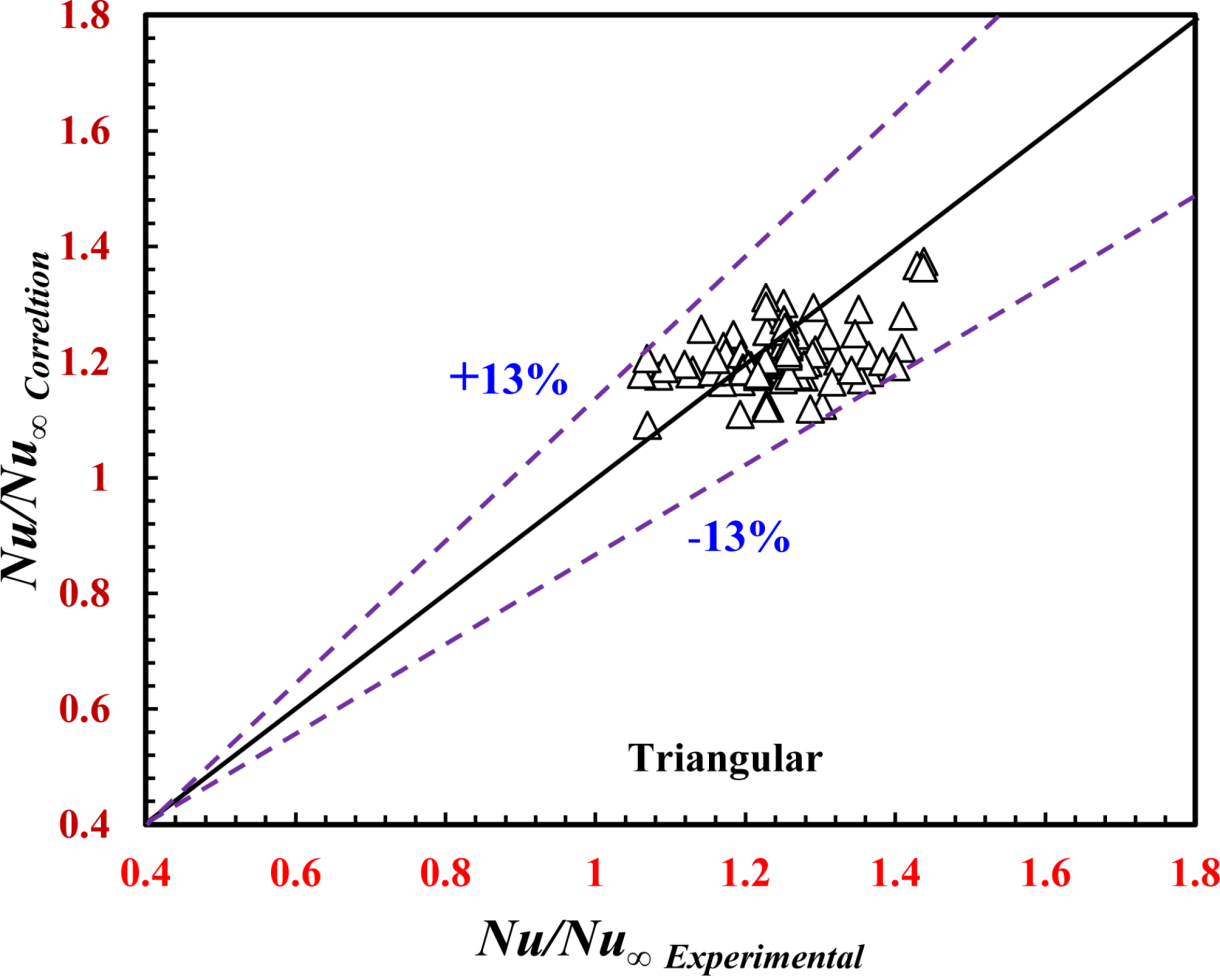
Verification of *Nu*_*o*_*/Nu*_*∞*_ for the present correlation and the experimental data.

**Fig. 15:**
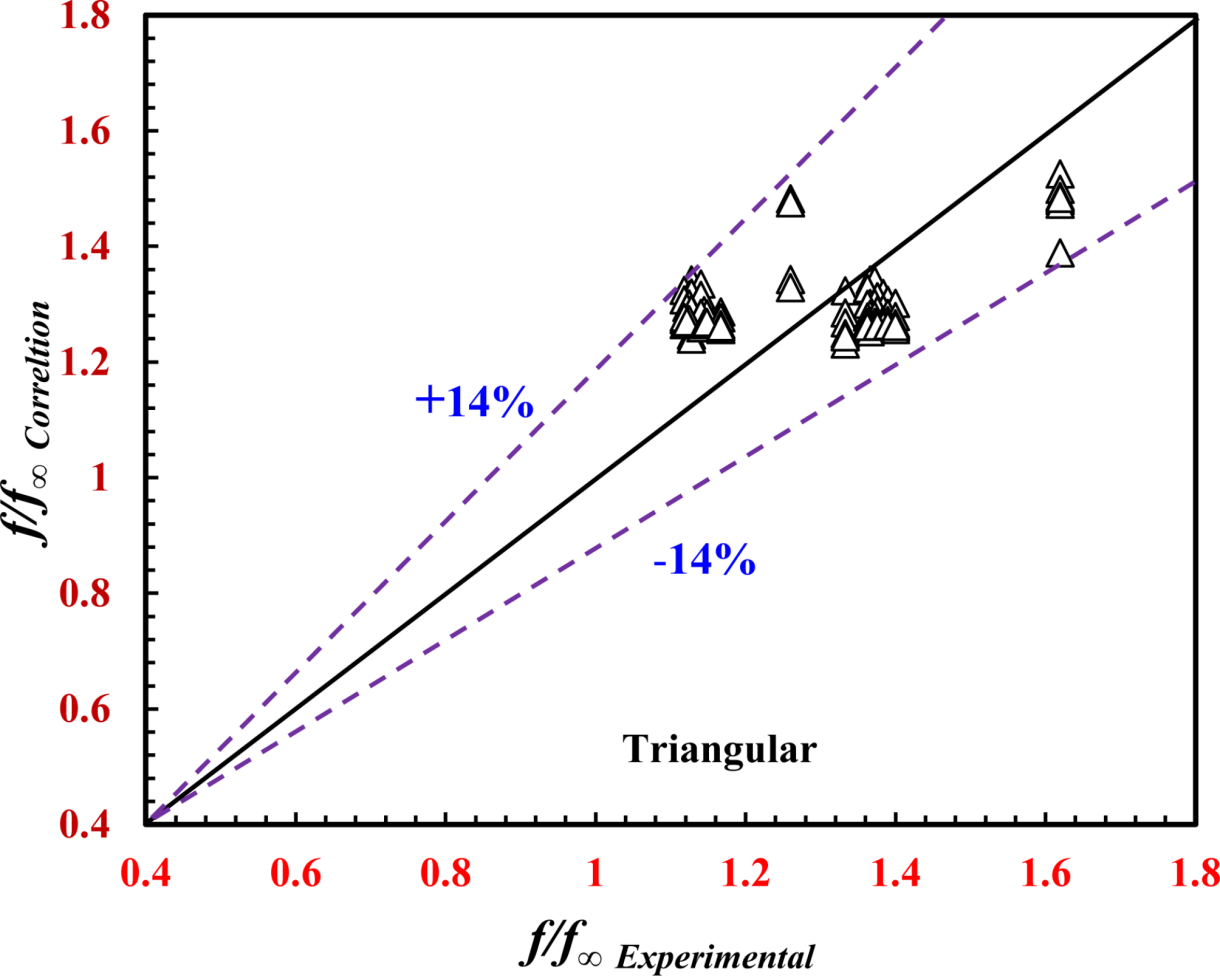
Verification of *f*_*o*_*/f*_*∞*_ for the present correlation and the experimental data.

Correlations (16) and (17) predict the present data with deviations of ± 13% and ± 14%, respectively, as shown in Figs. 14 and 15, and apply the following conditions:

$$2200 \le Re_{o} \le 17,700,{\mkern 1mu} {\mkern 1mu} 5.5 \le Pr_{o} \le 7.5,{\mkern 1mu} {\mkern 1mu} {\text{and}}{\mkern 1mu} 36{\text{~}}mm \le \Upsilon \le \infty {\mkern 1mu}$$

For the inner twisted square tube:

19 $${{N{u_o}} \mathord{\left/ {\vphantom {{N{u_o}} {N{u_\infty }}}} \right. \kern-0pt} {N{u_\infty }}}=0.0783Re_{o}^{{{\text{-0}}{\text{.207}}}}Pr_{o}^{{^{{{\text{2}}{\text{.45}}}}}}$$

20 $${{{f_o}} \mathord{\left/ {\vphantom {{{f_o}} {{f_\infty }}}} \right. \kern-0pt} {{f_\infty }}}={\text{1}}{\text{.109}}Re_{o}^{{0.0278}}$$

**Fig. 16:**
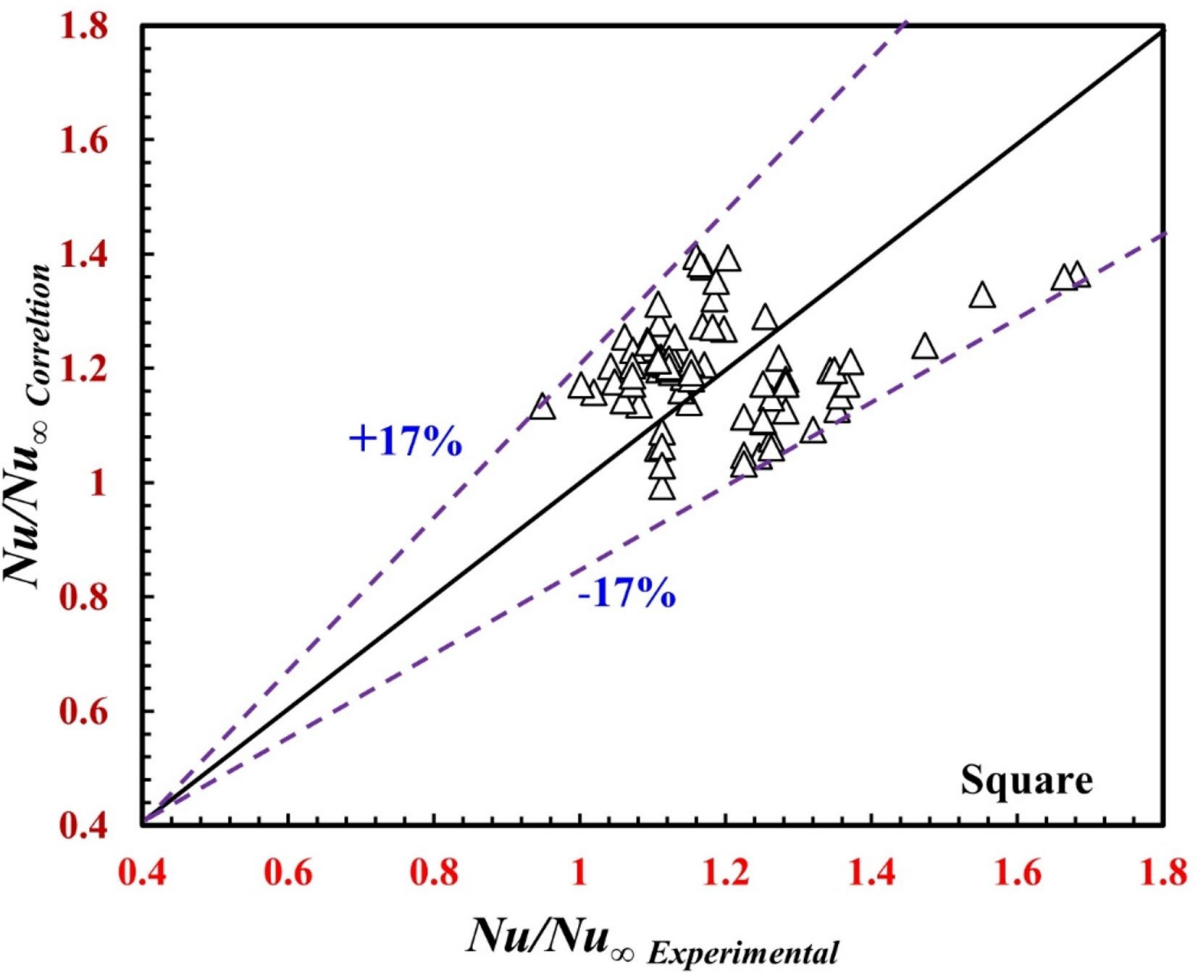
Verification of *Nu*_*o*_*/Nu*_*∞*_ for the present correlation and the experimental data.

**Fig. 17:**
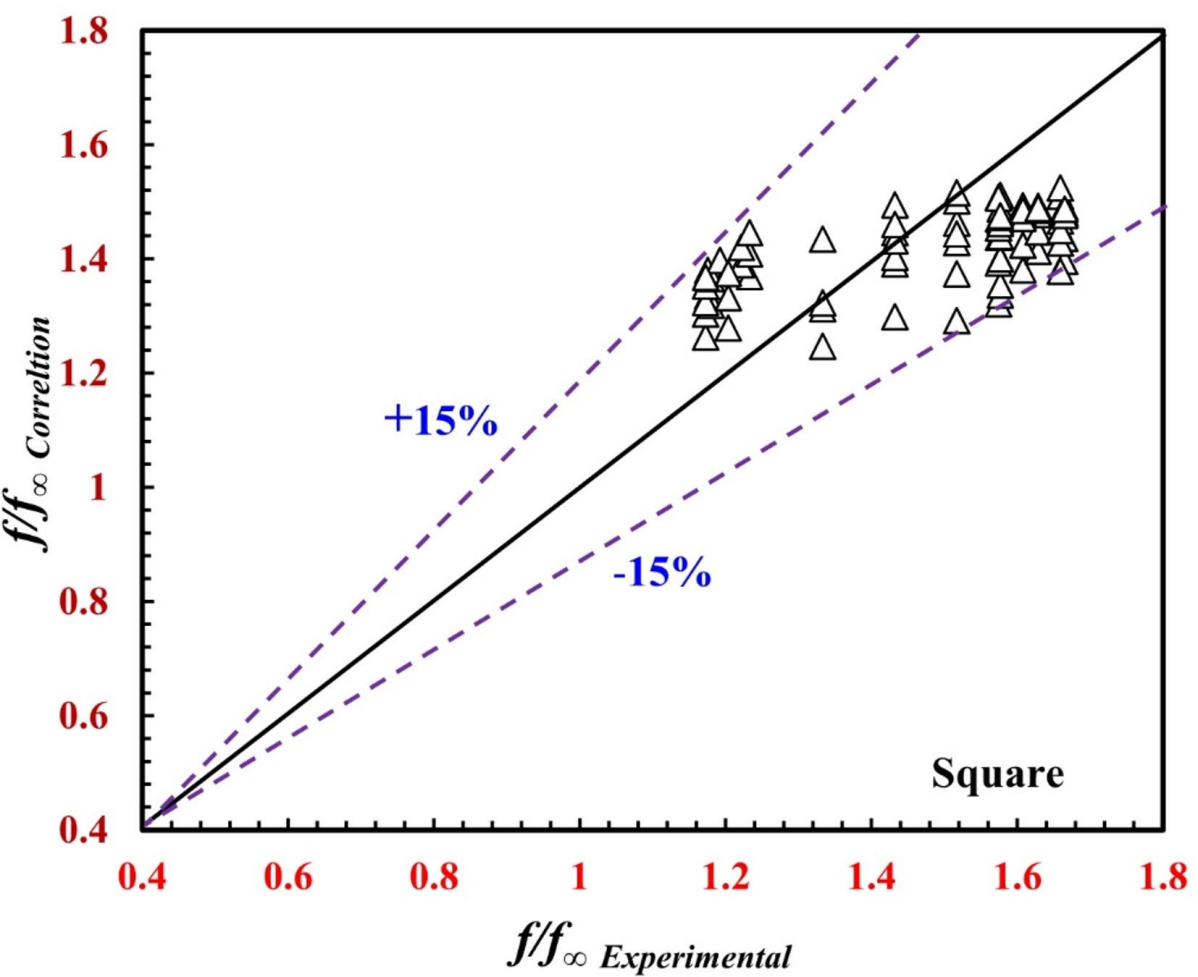
Verification of *f*_*o*_*/f*_∞_ for the present correlation and the experimental data.

Correlations (18) and (19) predict the present data with deviations of ± 17% and ± 15%, respectively, as shown in Figs. 16 and 17, and apply the following conditions:

$$2200 \le R{e_o} \le 17800,\,\,5.4 \le P{r_o} \le 7.5,\,\,{\text{and}}\,36 \; mm \le \,\Upsilon \le \infty .$$

## Conclusions

An experimental investigation was conducted to explore the thermal and exergetic characteristics of a *TTHC* in a counterflow arrangement. The effects of different twisted pitches of 36 mm, 54 mm, and ∞ (smooth/not twisted) on the thermofluid and exergetic characteristics with different cross-sectional profiles were presented. The main conclusions were drawn from the results as follows:

- The twisted tube enhances the heat transfer characteristics by destabilizing and detracting the thermal boundary layer.
- *Nu*_*o*_ increased with decreasing $\Upsilon$ to 36 mm and 54 mm compared to $\Upsilon$*=∞* (smooth tube/no twisted) by 39.6% and 24.3%, respectively, for the triangular inner twisted tube and 41.5% and 19.7%, respectively, for the square inner twisted tube.
- Increasing the number of twisted edges from three triangular edges to four square edges leads to increases in *Nu*_*o*_ for $\Upsilon$*=∞*,* 6*, and 9 of 13.1%, 7.3%, and 9.7%, respectively.
- _*Compared*_ with $\Upsilon$, fo increases by 37.6% and 14.5% for the triangular inner twisted tube and by 60.7% and 19.2%, respectively, for the square inner twisted tube designs.
- Increasing the number of inner twisted tube edges from three triangular edges to four square edges leads to decreases in *f*_*o*_ for $\Upsilon$*=∞*,* 6*, and 9 by 40.8%, 34.5%, and 21%, respectively.
- The maximum TPFs reached 1.3 and 1.25 for a $\Upsilon$ of 36 mm for triangular and square inner twisted tube shapes, respectively.
- Finally, the *TTHC* leads to a decrease in the exergy loss, *I*, for $\Upsilon$ = 36 mm and 54 mm compared to that for ∞ by 8% and 3.8%, respectively, for the triangular inner twisted tubes and by 9.4% and 3.7%, respectively, for the square inner twisted tubes.

## Data Availability

The datasets generated during and/or analyzed during the current study are available from corresponding author on reasonable request.

## List of symbols

*A*: Area, m^2^
*b*: Coil pitch, m
*C*: Specific heat, J kg^− 1^ °C^− 1^
*D*: Coil diameter, m
*d*_*h*_: Hydraulic diameter, m
*E*: Exergy, W
*f*: Darcy–Weisbach friction factor
*h*: Heat transfer coefficient, W m^− 2^ °C^−1^
*I*: Exergy destruction rate, (exergy loss), W
*k*: Thermal conductivity, W m^− 1^ °C^− 1^
*L*: Coil length, m
$\dot{m}$: Mass flow rate, kg s^− 1^
*Nu*: Nusselt number, (hd_h_/K)
*P*: Pressure, Pa
*Pr*: Prandtl number (µ Cp/k)
$\it \mathop Q\limits^{.}$: Heat transfer rate, W
*Re*: Reynolds number (ρvd_h_/µ)
*T*: Temperature, °C
*V*: Fluid velocity, m s^− 1^

## Greek symbols

*Δ*: Difference
*δ*: Curvature ratio
$\Upsilon$: Twisted tube pitch, m
*µ*: Dynamic viscosity, kg m^− 1^ s^− 1^
*η*_*EX.*_: Exergy efficiency

## Subscripts

*i*: Inner/inlet
*o*: Outer/outlet

## Abbreviations

*TTHC*: Twisted tube helical coil
